# Paralog-divergent Features May Help Reduce Off-target Effects of Drugs: Hints from Glucagon Subfamily Analysis

**DOI:** 10.1016/j.gpb.2017.03.004

**Published:** 2017-06-20

**Authors:** Zhining Sa, Jingqi Zhou, Yangyun Zou, Zhixi Su, Xun Gu

**Affiliations:** 1 State Key Laboratory of Genetic Engineering and MOE Key Laboratory of Contemporary Anthropology, School of Life Sciences, Fudan University, Shanghai 200433, China; 2 Department of Genetics, Development and Cell Biology, Program of Bioinformatics and Computational Biology, Iowa State University, Ames, IA 50011, USA

**Keywords:** Paralog, Functional divergence, Functional site, Drug specificity, Evolutionary conservation

## Abstract

Side effects from targeted drugs remain a serious concern. One reason is the nonselective binding of a drug to unintended proteins such as its **paralogs**, which are highly homologous in sequences and have similar structures and drug-binding pockets. To identify targetable differences between paralogs, we analyzed two types (type-I and type-II) of **functional divergence** between two paralogs in the known target protein receptor family G-protein coupled receptors (GPCRs) at the amino acid level. Paralogous protein receptors in glucagon-like subfamily, glucagon receptor (GCGR) and glucagon-like peptide-1 receptor (GLP-1R), exhibit divergence in ligands and are clinically validated drug targets for type 2 diabetes. Our data showed that type-II amino acids were significantly enriched in the binding sites of antagonist MK-0893 to GCGR, which had a radical shift in physicochemical properties between GCGR and GLP-1R. We also examined the role of type-I amino acids between GCGR and GLP-1R. The divergent features between GCGR and GLP-1R paralogs may be helpful in their discrimination, thus enabling the identification of binding sites to reduce undesirable side effects and increase the target specificity of drugs.

## Introduction

Precision medicine enables thedevelopment of targeted drugs and improvement of the therapeutic efficacy [1]. However, some targeted drugs are promiscuous, showing a high risk of severe side effects because they have unexpected targets and exhibit low specificity [2]. Cross-reactivity on protein paralogs may cause undesirable side effects of drugs [3]. Generated from gene duplications, paralogs are evolutionally homologous [4] and share similar protein sequences or structural features, thus comprising similar binding pockets with drugs. As a result, a drug that binds to the target protein encoded by one gene may also bind to its paralog [5].

Because most drug targets are paralogs [3], controlling target specificity to minimize side effects is required to create novel and safer drugs. Such control may be achieved by drug design guided by paralog-discriminating features, known as “selectivity filters” [3]. Therefore, identifying evolutionally-divergent features that enable paralog discrimination would be beneficial. It is well accepted that amino acids are evolutionally conserved if they are functionally important [6]. Therefore, an amino acid residue is said to be functionally or structurally important if it is evolutionally conserved [7], whereas an evolutionally-variable residue is said to be less important. It is thus believed that alterations in the evolutionary conservation at a particular residue imply that this residue may have been involved in the functional divergence of a gene family during the evolution [4].

Type-I functional divergence gives rise to the site-specific rate variation after gene duplication [8], [9]. Typically, an amino acid residue related to the type-I functional divergence is highly conserved in one duplicate gene, but highly variable in the other one. Drug binding sites tend to be functionally important. If a drug targets the conserved residue of type-I functionally-divergent site in one paralog, its binding to the non-conserved residue in another paralogs would be avoided. Therefore, the alteration in evolutionary conservation resulting from type-I functional divergence can distinguish one paralog from another, which may reduce the occurrence of cross-reactivity.

Type-II functional divergence brings about the change of site-specific property. Typically, amino acid residues are highly evolutionally conserved within each cluster of orthologous genes, *i.e.*, both residues play vital roles functionally or structurally for this gene family. However, a radical change of amino acid property at a homologous site occurred between the two duplicate genes. For example, one residue is positively-charged in a gene but its homologous residue in the duplicated gene is negatively-charged [10], [11]. If a drug is designed to be negatively-charged, it can bind to a positively-charged residue in one paralog, but not the negatively-charged one in another paralog. A shift in key physicochemical properties relevant to ligand binding interactions may result in alterations in binding features or affect the druggability of protein targets [12]. Therefore, type-II functional divergence features in physicochemical properties between paralogs can be exploited as selectivity filters to function as targetable differences [13].

The known target protein receptor family of G-protein coupled receptors (GPCRs) contributes significantly to side effects [14]. GPCRs constitute one of the largest families of membrane proteins with approximately 800 members encoded in the human genome [15]. According to the GRAFS classification system, GPCRs fall into five categories, including glutamate (G), rhodopsin (α, β, γ, and δ) (R), adhesion (A), frizzled/taste2 (F), and secretin (S) families [16]. It is estimated that 30%–40% of all drugs currently on the market target GPCRs [17]. Since the gene members of this superfamily arose from gene duplication [18], these gene targets are rich in paralogs.

In this study, aimed to reduce the side effects caused by paralogs, we tried to figure out the features that can tell one paralog apart from another in an evolutionary way. Glucagon receptor (GCGR) and glucagon-like peptide-1 receptor (GLP-1R), two clinically validated drug targets in patients with type 2 diabetes, were used as an example for functional divergence analysis. We illustrated the analytical pipeline to detect paralog-divergent features between GCGR and GLP-1R. We identified these features in the target-binding design of existing drugs such as the antagonist MK-0893 to GCGR, which can achieve target selectivity. The enrichment of type-II functionally-divergent residues in binding sites of MK-0893 to GCGR and the reduction of binding potency once transferring type-I functionally-divergent residue to another in GLP-1R, may imply the important role of type-II and type-I functional divergence between GCGR and GLP-1R in paralog discrimination, which may be useful for identifying binding sites to achieve target specificity and develop safer and more selective drugs. Residues related to functional divergence should be taken into account when conserved residues are considered as drug binding sites.

## Results and discussion

### Functional divergence between paralog GCGR and GLP-1R

The glucagon-like subfamily belongs to secretin type GPCRs and is rich in clinically validated targets [18]. This subfamily constitutes 4 hormone receptors duplicated from the early stage of vertebrates [19] (Figure S1). These receptors play crucial roles in hormonal homeostasis in humans and other animals and serve as important drug targets for several endocrine disorders [20]. Among them, GCGR and GLP-1R appear to have greater therapeutic potential in diabetes than other members [21], [22], [23]. Thus, we focused on GCGR and GLP-1R for further investigation.

GCGR shares high homology with GLP-1R, showing where 54% and 46% sequence identities in the transmembrane and extracellular domains, respectively [24], [25]. In addition, the corresponding ligands for GCGR and GLP-1R, glucagon and GLP-1, are also highly conserved in sequence [26]. It has been hypothesized that GLP-1 bound to GCGR and exhibited glucagon-like action in fish, but later it acquired unique incretin functions [27]. In humans, the tissue expression profile of *GCGR* and *GLP-1R*is different (Figure S2). *GCGR* is actively expressed in liver and kidney, whereas *GLP-1R* has relatively high expression in pancreas. This agrees with the fact that glucagon acts primarily on hepatic GCGR to increase plasma glucose, while GLP-1 functions during nutrient ingestion at pancreatic β-cell GLP-1R to enhance insulin synthesis and secretion [25]. These two hormones have significant but opposing roles in regulating glucose homeostasis and are clinically important in the management of diabetes [28]. GLP-1 affects blood glucose, β-cell protection, appetite, and body weight, which has led to the use of multiple GLP-1R agonists for the treatment of type 2 diabetes [29]. In contrast, glucagon is used to treat severe hypoglycemia [30], while GCGR antagonists have been developed to treat type 2 diabetes. Thus, GCGR and GLP-1R show divergent ligand binding profiles and are selective in hormone action, although they are highly homologous and show conserved structures and sequences. Therefore, when GCGR antagonists wrongly target highly homologous GLP-1R in patients with type 2 diabetes, these drugs may lose their efficacy and fail to control the release of glucose by GCGR. Moreover, the unexpected binding of these drugs to GLP-1R might interfere with function of GLP-1R, thus leading to the decreased insulin secretion. As a result, anti-diabetes drugs targeting one of these two paralogous receptors at conserved sites may also target the other one by mistake, resulting in cross-reactivity and generating unexpected side effects.

### Usage of type-II functional divergence features as targetable difference of drugs

#### Pipeline for type-II functional divergence analysis

**Figure 1:**
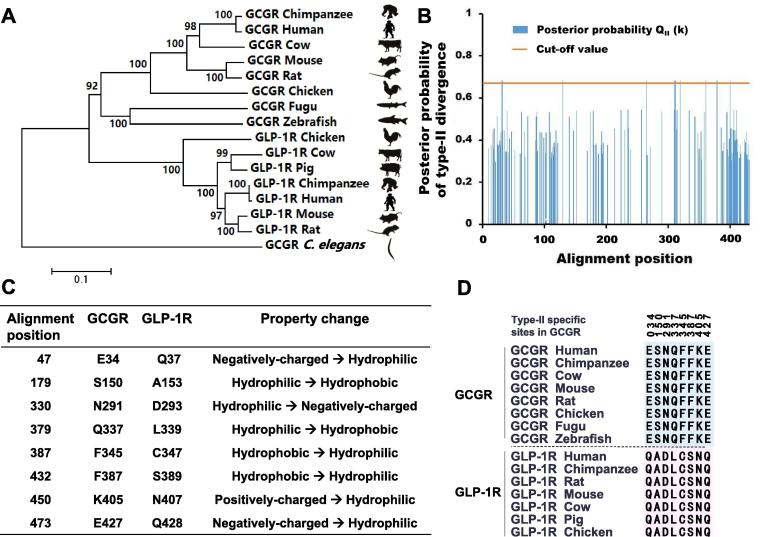
**Analytical pipeline for type-II functional divergence between GCGR and GLP-1R** **A.** Phylogenetic tree of GCGR and GLP-1R. **B.** Site-specific profile for predicting critical amino acid residues responsible for type-II functional divergence between GCGR and GLP-1R measured by posterior probability Q_II_ (k). **C.** Overview of amino acid changes in the eight predicted sites in type-II functional divergence. **D.** Sequence conservation analysis of the two clusters for GCGR and GLP-1R. GCGR, glucagon receptor; GLP-1R, glucagon-like peptide 1 receptor.

To avoid undesirable side effects driven by drug interactions with conserved residues of paralogs, we analyzed type-II functional divergence between GCGR and GLP-1R to identify residues conserved in functional constraints but different in physicochemical properties. A neighbor-joining tree was constructed to infer relationship between paralog GCGR and GLP-1R (Figure1A). Similar results were obtained when using other phylogenetic methods (*i.e.*, parsimony, maximum likelihood, and Bayesian methods; results not shown). The concordance of the results from different phylogenetic methods increased the confidence in the relationships inferred from the presented tree. Based on the phylogenetic tree, we estimated the coefficient of type-II functional divergence (denoted by θ_II_) between GCGR and GLP-1R. θ_II_ is 0.236 ± 0.052, which is significantly higher than 0 (*P* < 0.001). A large value of θ_II_ indicates a high level of type-II functional divergence, and *vice versa*. Rejection of the null hypothesis θ_II_ = 0 means that after gene duplication, the evolutionary rate has become different between the duplicate genes at some residues. Some amino acid residues that were evolutionally conserved in both GCGR and GLP-1R across different species may have radically changed their amino acid properties. Furthermore, we used the posterior probability Q_II_ (k) to identify amino acid residues critical in type-II functional divergence between these two paralogous genes (Figure1B). Using an empirical cutoff of Q_II_ (k) > 0.67 (posterior ratio R_II_ (k) > 2), we identified 8 type-II functional divergence-related residues between paralogous GCGR and GLP-1R. These included E34, S150, N291, Q337, F345, F387, K405, and E427 in GCGR. The site-specific ratio profile indicated that most residues had low posterior ratios and only a small portion of amino acid residues were involved in this type of functional divergence. Moreover, these 8 amino acid residues showed a typical pattern of type-II functional divergence (Figure1C). They showed a high sequence conservation at paralogous sites (Figure1D). We sized down the posterior probabilities of these sites and found that using lower posterior probability as cut-off value (such as 0.54) would screen out residues that were not presented in typical conservation pattern of type-II functional divergence (data not shown). Thus, we used these 8 type-II functional divergence-specific sites for further analysis about their roles in paralog discrimination.

#### Type-II functionally-divergent residues in binding sites of antidiabetic drugs

**Figure 2:**
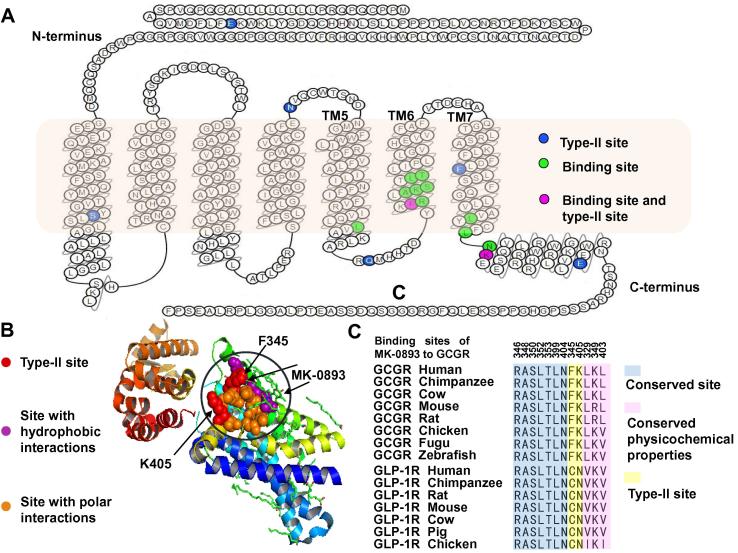
**Paralog-divergent features are considered targetable differences of drugs** **A.** Snake-plot diagram of GCGR with annotation of important residues. **B.** Different physicochemical properties of bipartite antagonist pocket corresponding to the dual polar/hydrophobic binding cleft in GCGR. **C.** Sequence conservation analysis of 12 binding sites of MK-0893 to GCGR.

The issues of cross-reactivity arising from paralogs have been long considered. Identifying paralog-divergent features as targetable difference might be helpful in paralog discrimination and has already been implemented in therapeutic drug design [31]. The GCGR antagonist MK-0893 is used to treat patients with type 2 diabetes to substantially reduce fasting and postprandial glucose concentrations [31]. MK-0893 acts at allosteric binding sites of the seven transmembrane helical domain (7TM) in positions among TM5, TM6, and TM7 in GCGR (Figure2A). TM6 plays a role in splitting the binding sites into two different interaction regions. The TM5-TM6 cleft contains L329, F345, L352, T353, and the alkyl chain of K349, making hydrophobic contacts with one part of MK-0893. On the other hand, the TM6-TM7 section forms polar interactions with the other part of MK-0893 by hydrogen bonds with K349, S350, L399, N404, and the backbone of K405, as well as additional salt bridge with R346. Thus, the different physicochemical properties function in the binding activity of the dual-nature antagonist MK-0893 to GCGR (Figure2B). We found that our predicted sites of type-II functional divergence between GCGR and GLP-1R, F345 and K405, were significantly enriched in the binding sites of MK-0893 to GCGR (*P* < 0.05; chi-square test).

To figure out the key difference in paralogous residues between GCGR and GLP-1R, we analyzed the sequence conservation in the binding sites of MK-0893 to GCGR and compared them with their equivalent sites in GLP-1R. The results showed that the type-II specific sites F345 and K405 had a radical shift in physicochemical properties, while other binding sites were highly conserved either in functional constraints or physicochemical properties between the two paralogs GCGR and GLP-1R (Figure2C). F345 and K405, showed a typical pattern of type-II functional divergence. They were both conserved residues in their orthologous gene families, but were different in their physicochemical properties between paralogous GCGR and GLP-1R. F345 was hydrophobic in GCGR but its equivalent site in GLP-1R is hydrophilic. If a molecule of drug is designed to be hydrophobic, it tends to bind to the hydrophobic F345 in GCGR rather than the hydrophilic residue in GLP-1R. Another type-II specific site K405 was positively-charged in GCGR while its equivalent site in GLP-1R was electrically neutral. Thus a molecule of drug designed to be negatively-charged are more likely to interact with positively-charged K405 in GCGR instead of binding to the electrically neutral residue in GLP-1R. Because the physiochemical properties of amino acids play an important role in the interaction of protein receptors with their ligands (small molecules, peptides, agonists, and antagonists), changes in their physicochemical nature and conformation may reduce cross-reactivity due to the binding of antagonist drugs to unexpected paralogs. Therefore, determining type-II functional divergence-related sites between two paralogs is effective for identifying targetable differences in therapeutic drug design.

Moreover, we investigated the binding of ligand and agonists to GLP-1R and evaluated the role of type-II functionally-divergent sites between GCGR and GLP-1R in this study. We identified a type-II functional divergence-related residue D293 within human GLP-1R in the second extracellular loop (EC2) (Table S1). D293 showed a typical pattern of type-II functional divergence. This residue is conserved in orthologous gene families of GLP-1R and is functionally important. It had ligand-specific effects on GLP-1 peptide-mediated selective signaling and was critical for agonist-mediated receptor activation [32]. Residue D293 of EC2 directly interacted with key residues in the ligand through hydrogen-bonding interactions (Table S1). A previous study [33] demonstrated that D293A mutation reduced GLP-1 affinity and altered the binding and efficacy of agonists such as oxyntomodulin and exendin-4 [34]. As a type-II functionally important site, D293 in GLP-1R showed different physicochemical properties from its equivalent site N291 in paralogous GCGR. The amino acid property changes from negatively-charged in GLP-1R to electrically neutral in GCGR, which can serve as a selective filter for telling apart GLP-1R from GCGR. Thus, the application of divergence features of type-II functional divergence between these two paralogs is advantageous in this respect.

### Using type-I functional divergence features as targetable difference of drugs

Besides type-II functional divergence, type-I functional divergence between paralogs might also be exploited to achieve targetable differences. We thus investigated the role of residues related to type-I functional divergence in the binding of ligand and agonists to GLP-1R. To do so, we computed the coefficient of type-I functional divergence (denoted by θ_I_; θ_I_ = 0 for null hypothesis) between GCGR and GLP-1R. We got θ_I_ value of 0.4902 ± 0.1072, which was significantly higher than 0 (*P* < 0.001), indicating the occurrence of type-I functional divergence between two paralogs. We identified a type-I-related residue E294 in the binding sites of GLP-1R (Table S1). E294 is a functionally important site for the signaling mechanism and receptor activation [32]. It is highly conserved in one cluster of orthologous GLP-1R family but appears as diverse amino acids at paralogous sites in GCGR. Therefore, the type-I functional divergence-related residues might play vital roles in drug binding sites for discrimination of two paralogs for tighter specificity control of drugs.

### Usage of variable residues as targetable difference of drugs

Not all binding sites of drugs have been designed to exploit the type-I or type-II functional divergence features as discriminating factors between paralogs. We therefore investigated more examples to see whether residues other than type-I or type-II functionally-divergent residues can achieve targetable difference between paralogs. We examined GCGR antagonist antibodies mAb1, mAb23, and mAb7 that target the ligand-binding cleft in the N-terminal extracellular domain, where the cleft is typically structurally important in ligand binding for secretin type GPCRs [35]. Our sequence conservation analysis of these antagonists illustrates that most binding-site residues showed significant conservation between paralogous GCGR and GLP-1R (*P* = 0.0003, 0.02, and 0.002 for mAb1, mAb23, and mAb7, respectively; chi-square test) (Table S2). Besides the most conserved residues, there are also some variable residues other than type-II or type-I specific residues in the binding sites. Mutations at these variable residues lead to structural differences such as a shift or changes in orientation of some side chain residues, thus resulting in reduced receptor activation and even prevention of ligand binding [36]. Therefore, these variable residues differ from one paralog to their equivalent sites in another paralog, while other residues in the binding sites are highly conserved either in sequence or in physicochemical properties. This implies that there may be underlying mechanisms involving variable residues in the discrimination of GCGR and GLP-1R.

### Identification of functional divergence of druggable paralogs in GPCRs

**Figure 3:**
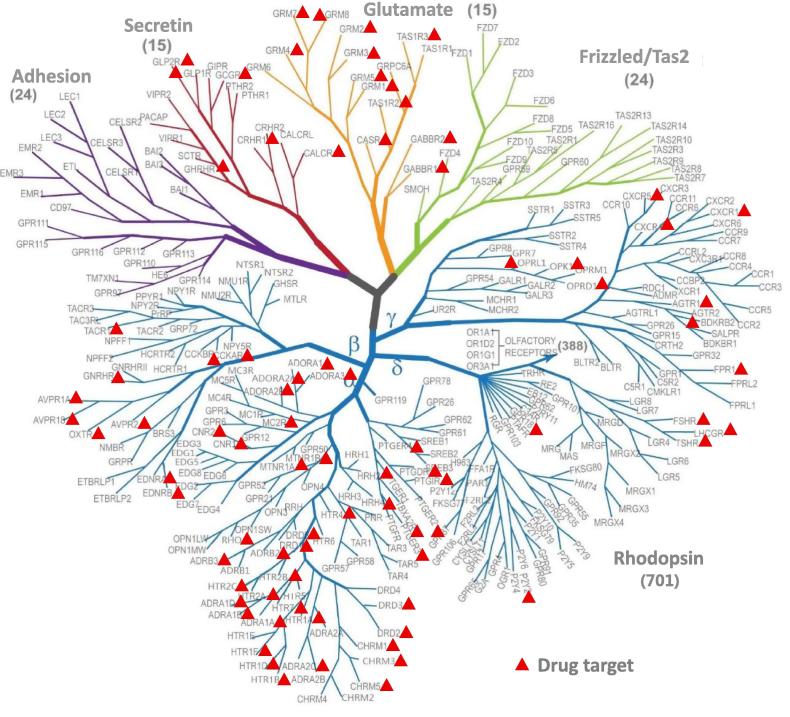
**Identification of drug targets in GPCRs** Rhodopsin, glutamate, and secretin subfamilies in GPCRs are abundant for drug targets. 83 targetable receptors are plotted on the GPCR tree (courtesy of Vsevolod Katritch and Raymond C Stevens from University of Southern California). GPCR, G-protein coupled receptor.

Inspired by the usage of functional divergence features in improving drug selectivity between paralog GCGR and GLP-1R, we hypothesized that these features might be applied to other paralogs of GPCRs in drug design. We thus extended to all targetable GPCRs and investigated their types of functional divergence between each paralogous gene pair. We identified 83 drug targets in total in GPCRs superfamily based on the published data on human druggable protein targets (Figure 3). We found that these targets are mainly enriched in rhodopsin, glutamate, and secretin subfamilies, which have been revealed to bind to various types of ligands and are targeted for drug design [17]. Among these 83 targets, 6 and 8 targets belong to the secretin and glutamate subfamilies, respectively, while others are found in 4 subgroups of rhodopsin subfamily. Interestingly, receptors in adhesion and frizzled/taste2 subfamilies are not found as drug targets. The majority of receptors in these two subfamilies remain orphans, and few attempts have been made to target these two classes.

Based on the two types of functional divergence between each paralogous pair in each subfamily, we found that, within 465 duplicated gene pairs, 267 pairs of paralogs have undergone functional divergence during the evolution. Among them, 67 pairs of paralogs showed only type-I functional divergence and 55 pairs showed only type-II functional divergence, whereas 145 pairs showed both two types of functional divergence (Table S3). Due to the lack of public data on drug binding sites for many targetable receptors in GPCRs family, we were not able to test the functional divergence features of all paralog pairs for verification. However, the site score for probability to be associated with type I or type II functional divergence is shown for each position on the multiple alignment of these paralogous gene pairs (Table S4). We systematically evaluated the large-scale functional divergence of each pair of paralogs in GPCRs to conclude the profiles of type-I or type-II related amino acid residues in every duplicated gene. These observations could be taken into consideration when designing conserved residues as drug binding sites (Table S5).

## Conclusion

Cross-reactivity arising from the structural and functional conservation of paralogs often results in undesirable side effects [37]. Although affinity toward these paralogs can be lower than that to the intended protein targets, the number of off-target paralogs could be sufficiently high to confer the side effects [38]. Given that, we came up with an evolutionary way to identify functional divergence features as paralog discrimination.

A detailed case study for the application of paralog-divergent features is exemplified to evaluate the roles of the two types of functional divergence in drug design. The type-II functional divergence-related residues are enriched in binding sites of MK-0893 to GCGR and become the only distinctive factor between paralogous GCGR and GLP-1R. This implies that taking the advantages of functional divergence features might be a choice to enhance paralog discrimination. These features have indeed been taken into account in living examples in drug design. Moreover, supported by other cases such as antagonist antibodies mAb1, mAb23, and mAb7 to GCGR, we concluded that most binding sites exhibit sequence conservation. Therefore, as type-I and type-II residues are highly correlated with conserved amino acid residues, more attention should be paid to their functional divergence features when considering conserved residues as drug binding sites, particularly when there are no variable residues in binding sites. Our study is further extended to the targetable genes in the whole GPCR family. We present the information of functional divergence-related residues, which may provide a point of reference for the selective binding of drugs to targetable receptors. With the more access of drug binding site data available, more usage of functional divergence features can improve drug selectivity and reduce side effects in the rational design of therapeutic drugs.

## Materials and methods

### Datasets

An evolutionary survey of druggable protein targets revealed 1632 genes as drug targets [39]. Canonically reciprocal best-to-best hits were considered to be 1:1 orthologs (human:macaque, human:mouse, and human:rat). Then 1362 genes in total belonging to an orthologous quartet (derived from the human, macaque, mouse, and rat genome) were given. We identified 83 druggable human GPCRs in total by data collection from the 1362 published gene targets and other literature research [40], [41]. We obtained 465 gene pairs of 83 targetable human GPCRs in the ENSEMBL database.

We downloaded 1312 amino acid sequences of targetable GPCRs and their paralogs in human as well as their vertebrate and invertebrate orthologs from the ENSEMBL database. To maintain uniqueness, partial and redundant sequences were removed, and only those genes with the longest proteins sequences were retained for further analysis.

### Multiple alignment and phylogenetic analysis

The multiple alignment of amino acid sequences was conducted using MEGA 7.0 software [42]. The alignment then was bootstrapped 500 times, providing a total 500 different alignments. To understand their relationship during evolution, we constructed phylogenetic trees for the paralogs in rhodopsin (α), rhodopsin (β), rhodopsin (γ), rhodopsin (δ), glutamate, and secretin subfamilies, respectively (Figure S3), which were inferred by the neighbor-joining method with Poisson distance. A phylogenetic tree of GCGR and GLP-1R was similarly constructed.

### Pipeline for functional divergence analysis

#### DIVERGE3.0: Posterior analysis to predict functionally-divergent residues

DIVERGE3.0 [43] was used to explore the functional evolution of druggable GPCRs family sequences. The site-specific profiles of every two duplicate gene clusters were determined to detect amino acid residues that are crucial for the type (I or II) of functional divergence. Posterior ratios are defined as R_I_ (k) = Q_I_ (k)/[1 − Q_I_ (k)] and R_II_ (k) = Q_II_ (k)/[1 − Q_II_ (k)], where Q_I_ (k) and Q_II_ (k) refer to the posterior probability at site k for type I and type-II functional divergence, respectively. Under a given empirical cut-off value, we screened important residues related to type-I or type-II functional divergence between duplicated genes.

#### Type-I and type-II functional divergence

The probability of a residue being under functional divergence-related state is denoted by θ. θ is estimated by the maximum likelihood method proposed by Gu [9] and calculated in *DIVERGE3.0.* θ_I_ or θ_II_ is the coefficient that measures the probability of type-I or type-II functional divergence between duplicate genes. Larger value of θ_I_ or θ_II_ indicates the higher level of involvement in type-I or type-II functional divergence, and *vice versa*. After the event of gene duplication, the coefficient of type-I or type-II functional divergence between two duplicate genes can be estimated [44]. Rejection of the null hypothesis θ_I_ = 0 or θ_II_ = 0 indicates the occurrence of shifts in the evolutionary rate at some sites between two duplicate genes. For the type-II functional divergence, amino acids are classified into four groups [45]: positively-charged (K, R, and H), negatively-charged (D and E), hydrophilic (S, T, N, Q, C, G, and P), and hydrophobic (A, I, L, M, F, W, V, and Y). When an amino acid changes from one group to another, it is referred to as radical; otherwise, it is conserved.

### Mapping predicted amino acid residues to protein structure

Snake-plot diagrams produced by web tools in the GPCRdb database [46] were used to represent seven transmembrane helical structure of GCGR.

Crystal structure of the human GCGR chain A in complex with the antagonist MK-0893 was downloaded from RCSB Protein Data Bank (PDB ID 5EE7) [47]. PyMOL software (Schrodinger, LLC) was then used to illustrate the roles of physicochemical properties in target binding events and to understand the importance of different physicochemical features of functional divergence-related residues at the equivalent sites between two paralogs in setting one paralog apart from another for drugs binding.

## Authors’ contributions

ZNS carried out the evolutionary studies of GPCR paralogs using DIVERGE3.0 software and functional analysis of important amino acids in drug binding sites. JZ participated in the large-scale investigation of amino acids sites under functional divergence between paralogs of targetable genes in GPCRs. YZ participated in the study design. ZXS provided advices for binding sites analysis. XG conceived of the study and provided idea for functional divergence and raw data of druggable genes. ZNS drafted the manuscript with the help from YZ and XG. All authors read and approved the final manuscript.

## Competing interests

The authors have declared no competing interests.

## Supplementary material

Supplementary Figure S1 **Phylogenetic tree of glucagon-like subfamily**The tree reflects the evolutionary history of members of glucagon-like subfamily of GPCRs, which are rich for validated drug targets, consisting of GCGR, GLP1R, GLP2R (drug targets), and GIPR (non-drug target). GCGR, glucagon receptor; GLP-1R, glucagon-like peptide 1 receptor; GLP-2R, glucagon-like peptide 2 receptor; GIPR, gastric inhibitory polypeptide receptor; GHRHR, growth-hormone-releasing hormone receptor; VIPR1, vasoactive intestinal peptide receptor 1; SCTR, secretin receptor; CALCR, calcitonin receptor.

Supplementary Figure S2 **Expression analysis of the paralogous genes *GCGR* and *GLP-1R***The expression levels (RPKM) in human tissues between gene *GCGR* (**A**) and *GLP-1R* (**B**) are presented as box plots. *GCGR* are highly expressed in kidney and liver, while *GLP-1R* are more likely to be expressed in other tissues such as pancreas, heart, brain, and stomach, except for kidney and liver (data source: GTEx Analysis Release V6p). RPKM, reads per kilobase of transcript per million mapped reads.

Supplementary Figure S3 **Phylogenetic trees of paralogs in druggable rhodopsin (α), rhodopsin (β), rhodopsin (γ), rhodopsin (δ), glutamate, and secretin subfamiliesA.** The α group of rhodopsin family is formed by the prostaglandin receptor cluster, amine receptor cluster, melatonin receptor cluster, opsin receptor cluster, and MECA receptor cluster. **B.** The β group of rhodopsin family is mainly formed by peptide receptor cluster. **C.** The γ group of rhodopsin family is formed by SOGR cluster, MCHR cluster, and chemokine receptor cluster. **D.** The δ group of rhodopsin family is formed by MAS-related receptor cluster, glycoprotein receptor cluster, purine receptor cluster, and olfactory receptor cluster. **E.** The glutamate family is mainly composed of GRM, GABA receptors, CASR, TAS1Rs, as well as other orphan receptors. **F.** The GABBR1 and GABBR2 are a pair of targetable paralogs in glutamate subfamily. **G.** The receptors in secretin family include VIPRs, GHRHRs, SCTRs, GCGRs, GLP-1R and GLP-2R, GIPRs, CRHRs and parathyroid hormone receptor (PTHRs). GRM, metabotropic glutamate receptor; MECA, melanocortin, endothelin, cannabinoid, and adenosine; SOGR, somatostatin, opioid, and galanin receptor; MCHR, melanin-concentrating hormone receptor; GABA, gamma aminobutyric acid; GCGR, glucagon receptor; VIPR1, vasoactive intestinal peptide receptor 1; VIPR2, vasoactive intestinal peptide receptor 2; PACAPR, pituitary adenylate cyclase-activating polypeptide receptor; GHRHR, growth hormone releasing hormone receptor; GLPR, glucagon-related peptide receptor; GIPR, gastric inhibitory polypeptide receptor; CRFR, corticotropin releasing factor receptor; CASR, calcium-sensing receptor; TAS1R, sweet and umami taste receptor; SCTR, secretin receptor; CALCR, calcitonin receptor.

Supplementary Table S1 **Type-I and type-II functionally-divergent residues in the binding sites of ligand and agonists to GLP-1R**

Supplementary Table S2 **Variable residues in the binding sites of antagonist antibodies to GCGR**

Supplementary Table S3 **Overview of functional divergence among paralogs of druggable members in GPCRs**

Supplementary Table S4 **The site-specific profiles of paralogs in druggable subfamilies of GPCRs**

Supplementary Table S5 **Identification of important amino acid residues related to type-I or type-II functional divergence**
