# Supplementary material for: Paralog-divergent Features May Help Reduce Off-target Effects of Drugs: Hints from Glucagon Subfamily Analysis
Source: Genomics Proteomics Bioinformatics. 2017 Jun 20;15(4):246–54. doi: 10.1016/j.gpb.2017.03.004 (PMC5582795; doi:10.1016/j.gpb.2017.03.004)
Supplement: Supplementary Figure S1 — Phylogenetic tree of glucagon-like subfamilyThe tree reflects the evolutionary history of members of glucagon-like subfamily of GPCRs, which are rich for validated drug targets, consisting of GCGR, GLP1R, GLP2R (drug targets), and GIPR (non-drug target). GCGR, glucagon receptor; GLP-1R, glucagon-like peptide 1 receptor; GLP-2R, glucagon-like peptide 2 receptor; GIPR, gastric inhibitory polypeptide receptor; GHRHR, growth-hormone-releasing hormone receptor; VIPR1, vasoactive intestinal peptide receptor 1; SCTR, secretin receptor; CALCR, calcitonin receptor. [file mmc1.pptx]

## Slide 1
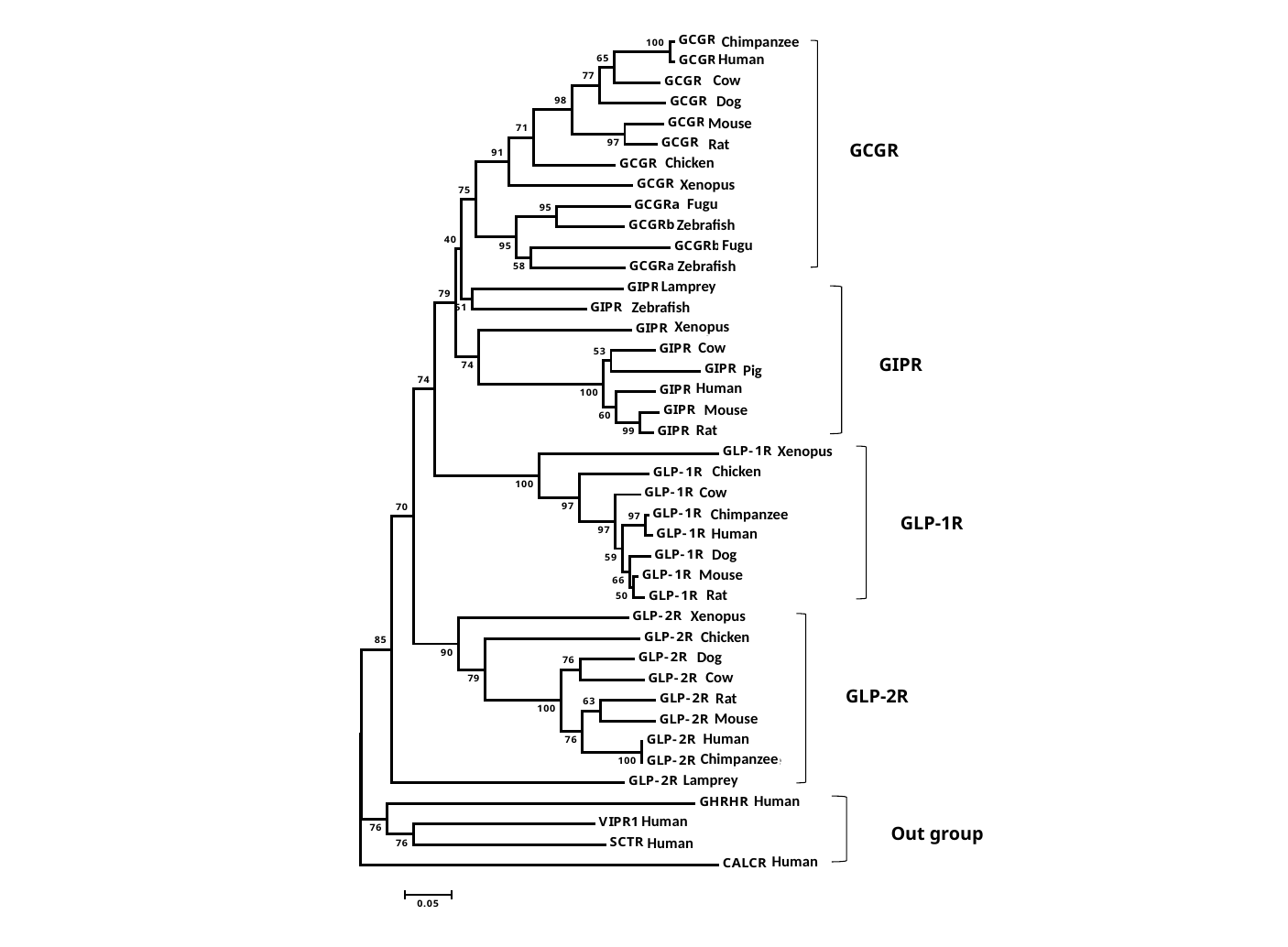

GCGR
GIPR
GLP-1R
GLP-2R
Out group
Chimpanzee
Human
Cow
Dog
Mouse
Rat
Chicken
Xenopus
Fugu
Zebrafish
Fugu
Zebrafish
Lamprey
Zebrafish
Xenopus
Cow
Pig
Human
Mouse
Rat
Xenopus
Chicken
Cow
Chimpanzee
Human
Dog
Mouse
Rat
Xenopus
Chicken
Dog
Cow
Rat
Mouse
Human
Chimpanzee
Lamprey
Human
Human
Human
Human
