# Supplementary material for: Paralog-divergent Features May Help Reduce Off-target Effects of Drugs: Hints from Glucagon Subfamily Analysis
Source: Genomics Proteomics Bioinformatics. 2017 Jun 20;15(4):246–54. doi: 10.1016/j.gpb.2017.03.004 (PMC5582795; doi:10.1016/j.gpb.2017.03.004)
Supplement: Supplementary Figure S2 — Expression analysis of the paralogous genes GCGR and GLP-1RThe expression levels (RPKM) in human tissues between gene GCGR (A) and GLP-1R (B) are presented as box plots. GCGR are highly expressed in kidney and liver, while GLP-1R are more likely to be expressed in other tissues such as pancreas, heart, brain, and stomach, except for kidney and liver (data source: GTEx Analysis Release V6p). RPKM, reads per kilobase of transcript per million mapped reads. [file mmc2.pptx]

## Slide 1
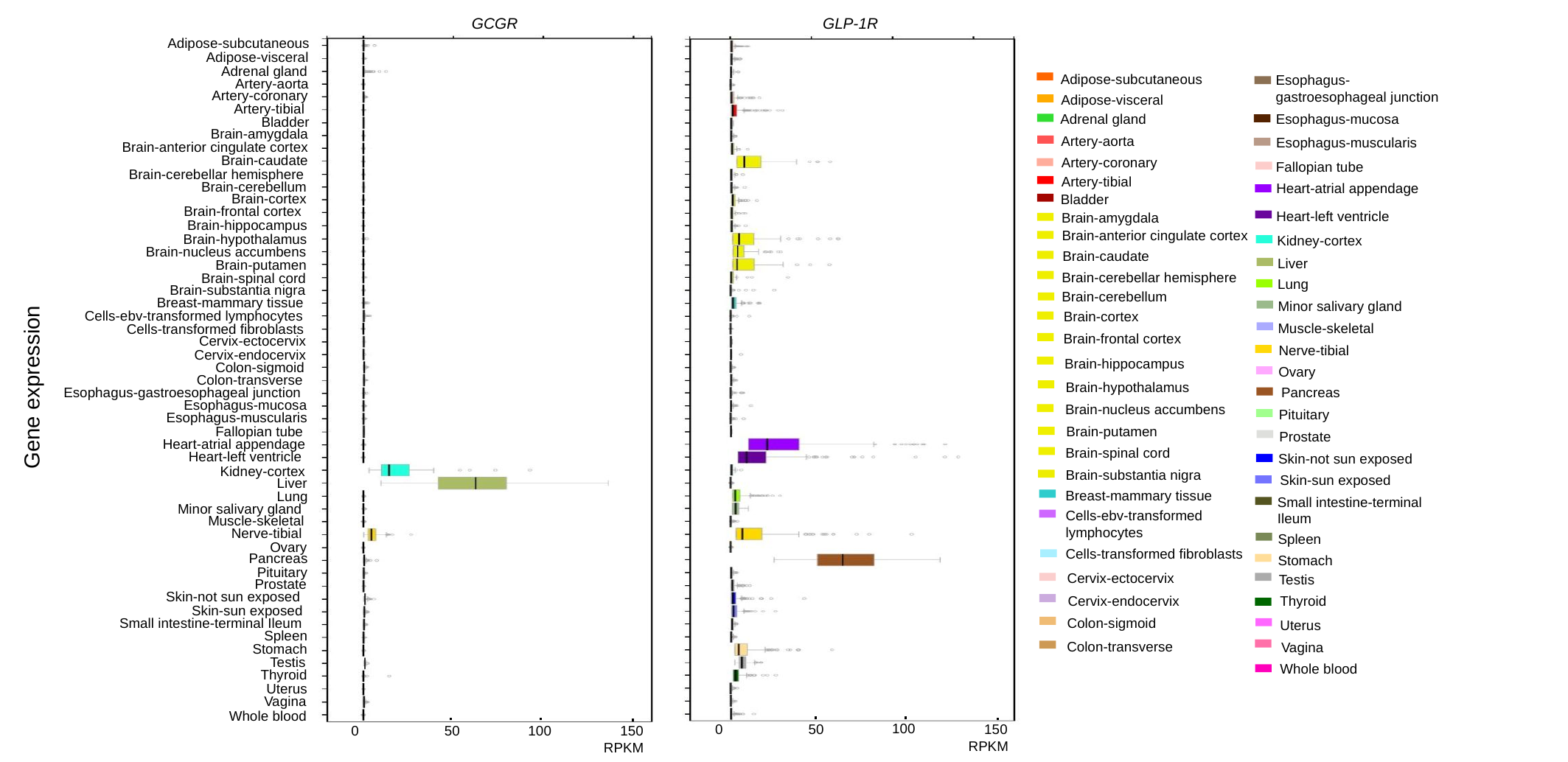

GCGR
GLP-1R
Adipose-subcutaneous
Adipose-visceral
Adrenal gland
Artery-aorta
Artery-coronary
Artery-tibial
Bladder
Brain-amygdala
Brain-anterior cingulate cortex
Brain-caudate
Brain-cerebellar hemisphere
Brain-cerebellum
Brain-cortex
Brain-frontal cortex
Brain-hippocampus
Brain-hypothalamus
Brain-nucleus accumbens
Brain-putamen
Brain-spinal cord
Brain-substantia nigra
Breast-mammary tissue
Cells-ebv-transformed lymphocytes
Cells-transformed fibroblasts
Cervix-ectocervix
Cervix-endocervix
Colon-sigmoid
Colon-transverse
Esophagus-gastroesophageal junction
Esophagus-mucosa
Esophagus-muscularis
Fallopian tube
Heart-atrial appendage
Heart-left ventricle
Kidney-cortex
Liver
Lung
Minor salivary gland
Muscle-skeletal
Nerve-tibial
Ovary
Pancreas
Pituitary
Prostate
Skin-not sun exposed
Skin-sun exposed
Small intestine-terminal Ileum
Spleen
Stomach
Testis
Thyroid
Uterus
Vagina
Whole blood
Adipose-subcutaneous
Esophagus-
gastroesophageal junction
Adipose-visceral
Adrenal gland
Esophagus-mucosa
Artery-aorta
Esophagus-muscularis
Artery-coronary
Fallopian tube
Artery-tibial
Heart-atrial appendage
Bladder
Heart-left ventricle
Brain-amygdala
Brain-anterior cingulate cortex
Kidney-cortex
Brain-caudate
Liver
Brain-cerebellar hemisphere
Lung
Brain-cerebellum
Minor salivary gland
Brain-cortex
Muscle-skeletal
Brain-frontal cortex
Nerve-tibial
Brain-hippocampus
Ovary
Brain-hypothalamus
Pancreas
Brain-nucleus accumbens
Pituitary
Brain-putamen
Prostate
Brain-spinal cord
Skin-not sun exposed
Brain-substantia nigra
Skin-sun exposed
Breast-mammary tissue
Small intestine-terminal
Ileum
Cells-ebv-transformed
lymphocytes
Spleen
Cells-transformed fibroblasts
Stomach
Cervix-ectocervix
Testis
Cervix-endocervix
Thyroid
Colon-sigmoid
Uterus
Colon-transverse
Vagina
Whole blood
Gene expression
100
0
150
50
100
0
150
50
RPKM
RPKM
