# Supplementary material for: Paralog-divergent Features May Help Reduce Off-target Effects of Drugs: Hints from Glucagon Subfamily Analysis
Source: Genomics Proteomics Bioinformatics. 2017 Jun 20;15(4):246–54. doi: 10.1016/j.gpb.2017.03.004 (PMC5582795; doi:10.1016/j.gpb.2017.03.004)
Supplement: Supplementary Figure S3 — Phylogenetic trees of paralogs in druggable rhodopsin (α), rhodopsin (β), rhodopsin (γ), rhodopsin (δ), glutamate, and secretin subfamiliesA. The α group of rhodopsin family is formed by the prostaglandin receptor cluster, amine receptor cluster, melatonin receptor cluster, opsin receptor cluster, and MECA receptor cluster. B. The β group of rhodopsin family is mainly formed by peptide receptor cluster. C. The γ group of rhodopsin family is formed by SOGR cluster, MCHR cluster, and chemokine receptor cluster. D. The δ group of rhodopsin family is formed by MAS-related receptor cluster, glycoprotein receptor cluster, purine receptor cluster, and olfactory receptor cluster. E. The glutamate family is mainly composed of GRM, GABA receptors, CASR, TAS1Rs, as well as other orphan receptors. F. The GABBR1 and GABBR2 are a pair of targetable paralogs in glutamate subfamily. G. The receptors in secretin family include VIPRs, GHRHRs, SCTRs, GCGRs, GLP-1R and GLP-2R, GIPRs, CRHRs and parathyroid hormone receptor (PTHRs). GRM, metabotropic glutamate receptor; MECA, melanocortin, endothelin, cannabinoid, and adenosine; SOGR, somatostatin, opioid, and galanin receptor; MCHR, melanin-concentrating hormone receptor; GABA, gamma aminobutyric acid; GCGR, glucagon receptor; VIPR1, vasoactive intestinal peptide receptor 1; VIPR2, vasoactive intestinal peptide receptor 2; PACAPR, pituitary adenylate cyclase-activating polypeptide receptor; GHRHR, growth hormone releasing hormone receptor; GLPR, glucagon-related peptide receptor; GIPR, gastric inhibitory polypeptide receptor; CRFR, corticotropin releasing factor receptor; CASR, calcium-sensing receptor; TAS1R, sweet and umami taste receptor; SCTR, secretin receptor; CALCR, calcitonin receptor. [file mmc3.pptx]

## Slide 1
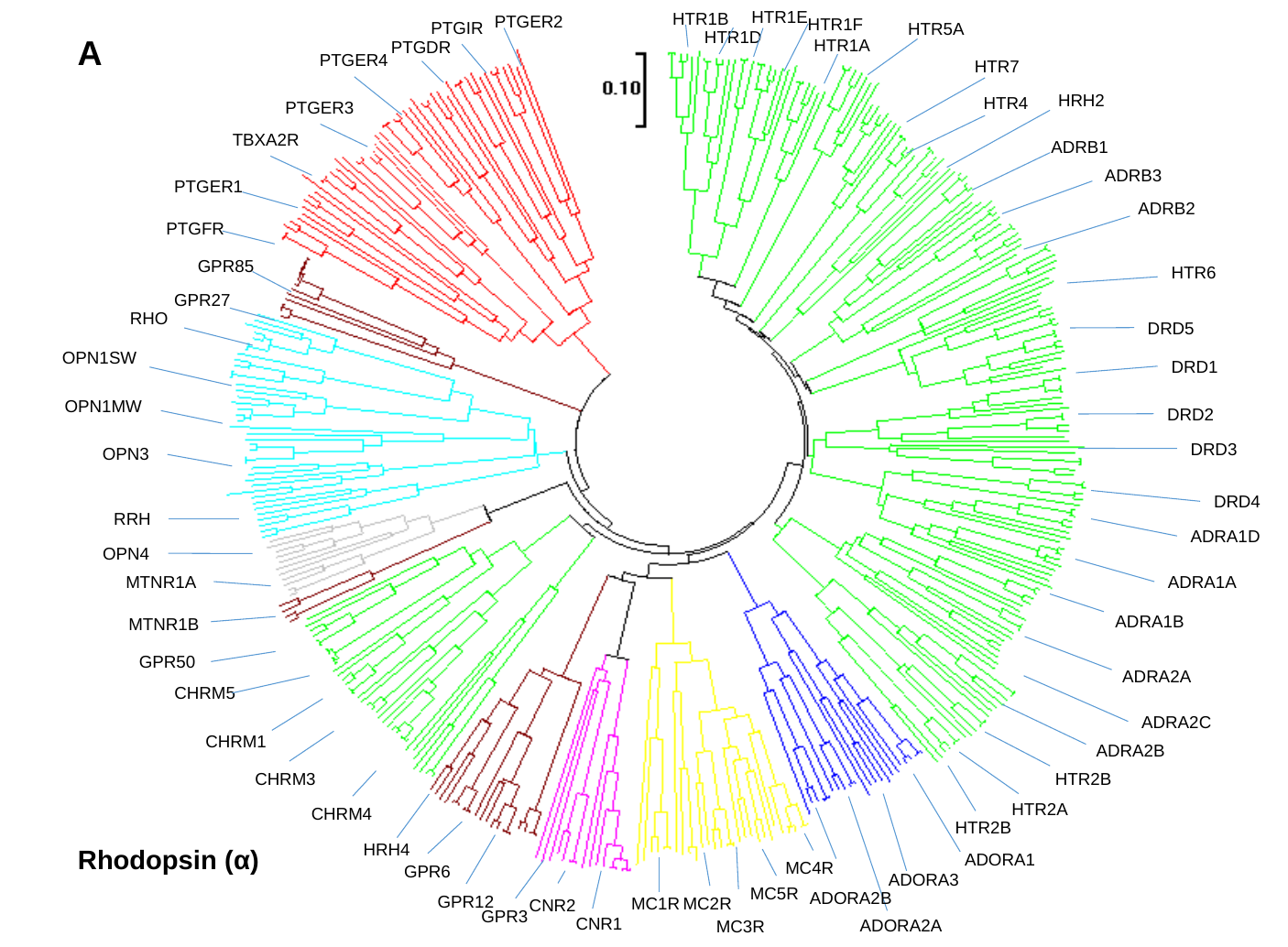

HTR1E
HTR1B
PTGER2
HTR1F
PTGIR
HTR5A
HTR1D
A
HTR1A
PTGDR
PTGER4
HTR7
HRH2
HTR4
PTGER3
TBXA2R
ADRB1
ADRB3
PTGER1
ADRB2
PTGFR
GPR85
HTR6
GPR27
RHO
DRD5
OPN1SW
DRD1
OPN1MW
DRD2
DRD3
OPN3
DRD4
RRH
ADRA1D
OPN4
MTNR1A
ADRA1A
ADRA1B
MTNR1B
GPR50
ADRA2A
CHRM5
ADRA2C
CHRM1
ADRA2B
HTR2B
CHRM3
HTR2A
CHRM4
HTR2B
HRH4
Rhodopsin (α)
ADORA1
MC4R
GPR6
ADORA3
MC5R
ADORA2B
GPR12
MC1R
MC2R
CNR2
GPR3
CNR1
ADORA2A
MC3R

## Slide 2
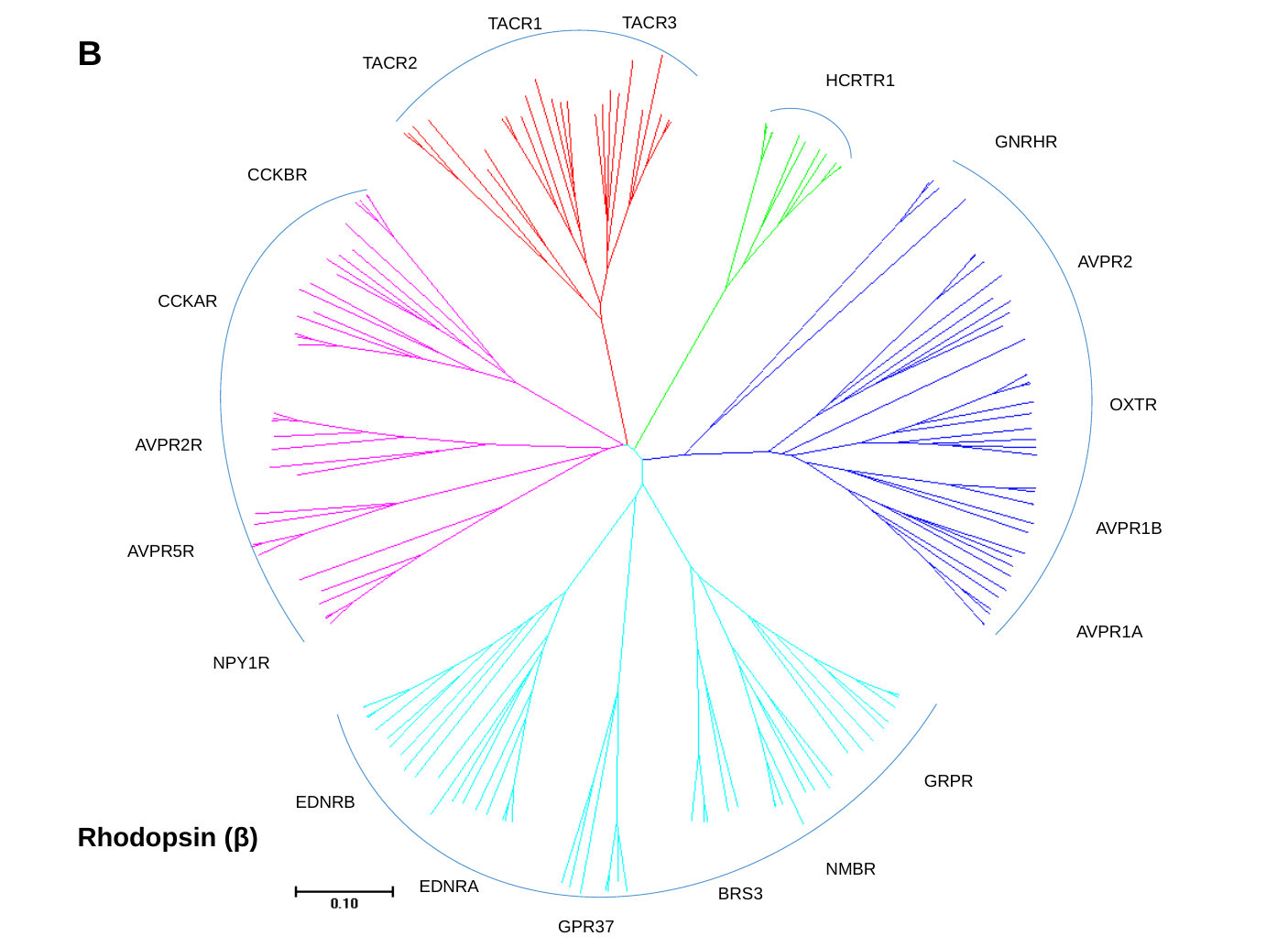

TACR3
TACR1
B
TACR2
HCRTR1
GNRHR
CCKBR
AVPR2
CCKAR
OXTR
AVPR2R
AVPR1B
AVPR5R
AVPR1A
NPY1R
GRPR
EDNRB
Rhodopsin (β)
NMBR
EDNRA
BRS3
GPR37

## Slide 3
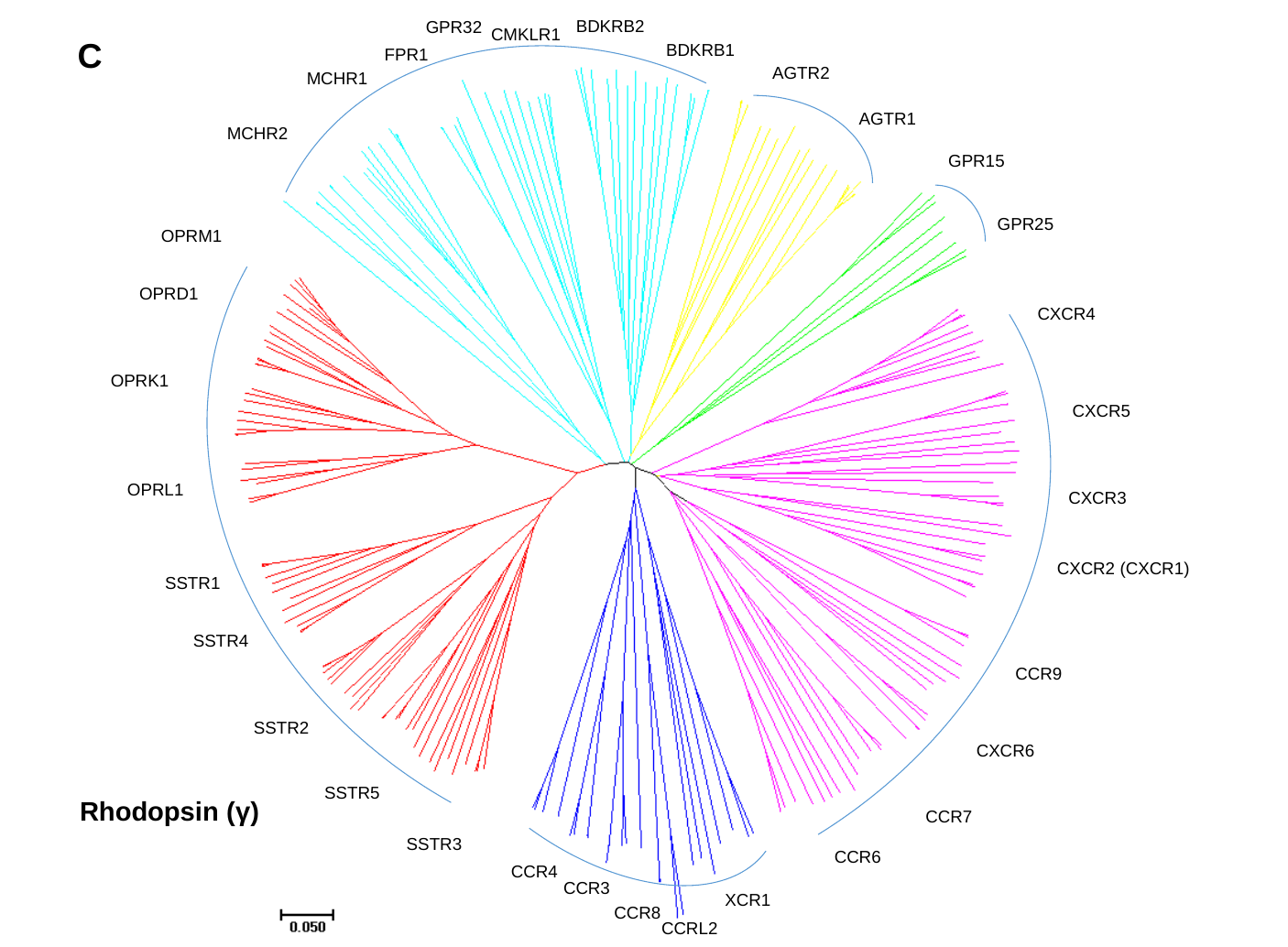

BDKRB2
GPR32
CMKLR1
C
BDKRB1
FPR1
AGTR2
MCHR1
AGTR1
MCHR2
GPR15
GPR25
OPRM1
OPRD1
CXCR4
OPRK1
CXCR5
OPRL1
CXCR3
CXCR2 (CXCR1)
SSTR1
SSTR4
CCR9
SSTR2
CXCR6
SSTR5
Rhodopsin (γ)
CCR7
SSTR3
CCR6
CCR4
CCR3
XCR1
CCR8
CCRL2

## Slide 4
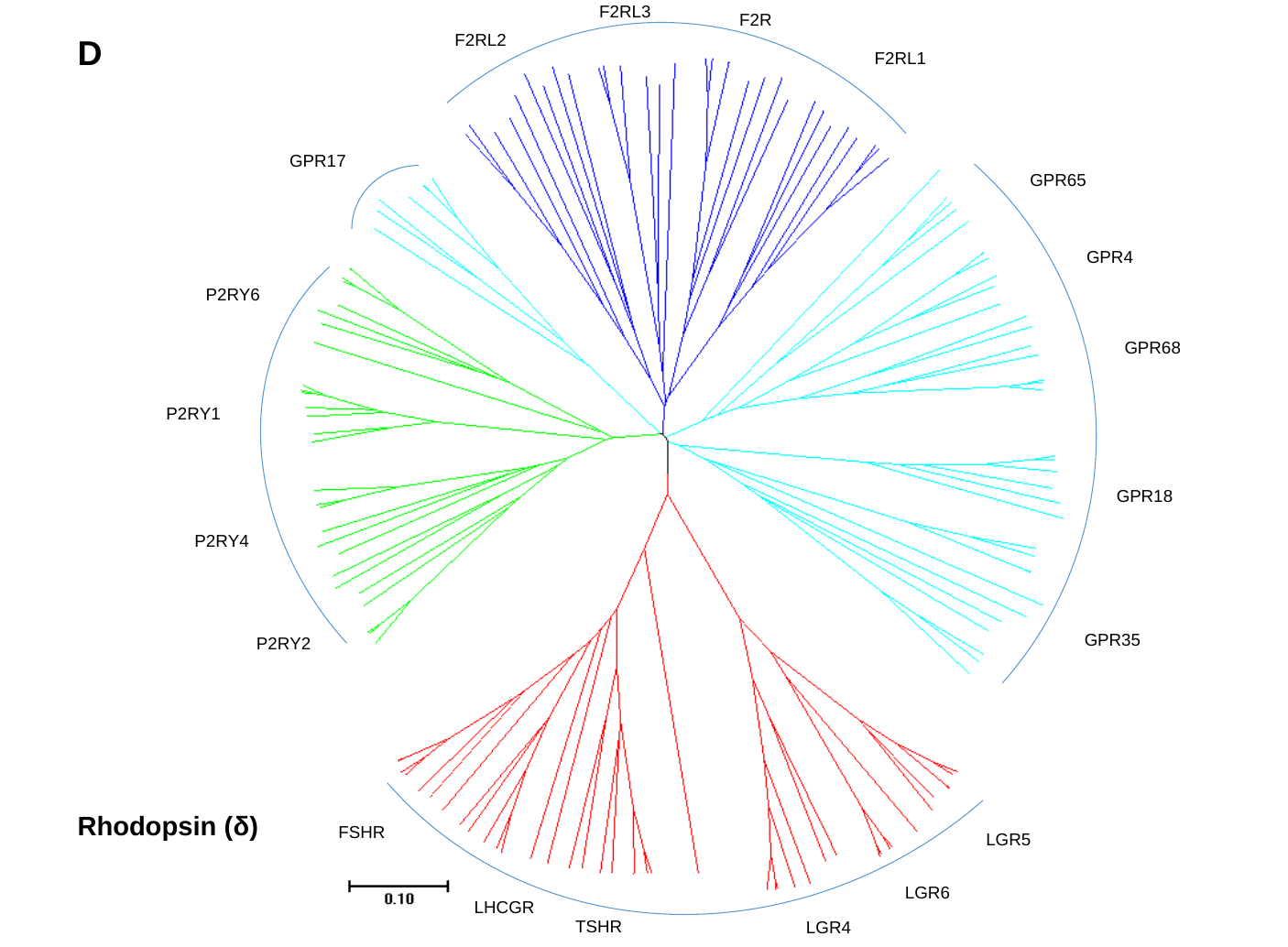

F2RL3
F2R
F2RL2
D
F2RL1
GPR17
GPR65
GPR4
P2RY6
GPR68
P2RY1
GPR18
P2RY4
GPR35
P2RY2
Rhodopsin (δ)
FSHR
LGR5
LGR6
LHCGR
TSHR
LGR4

## Slide 5
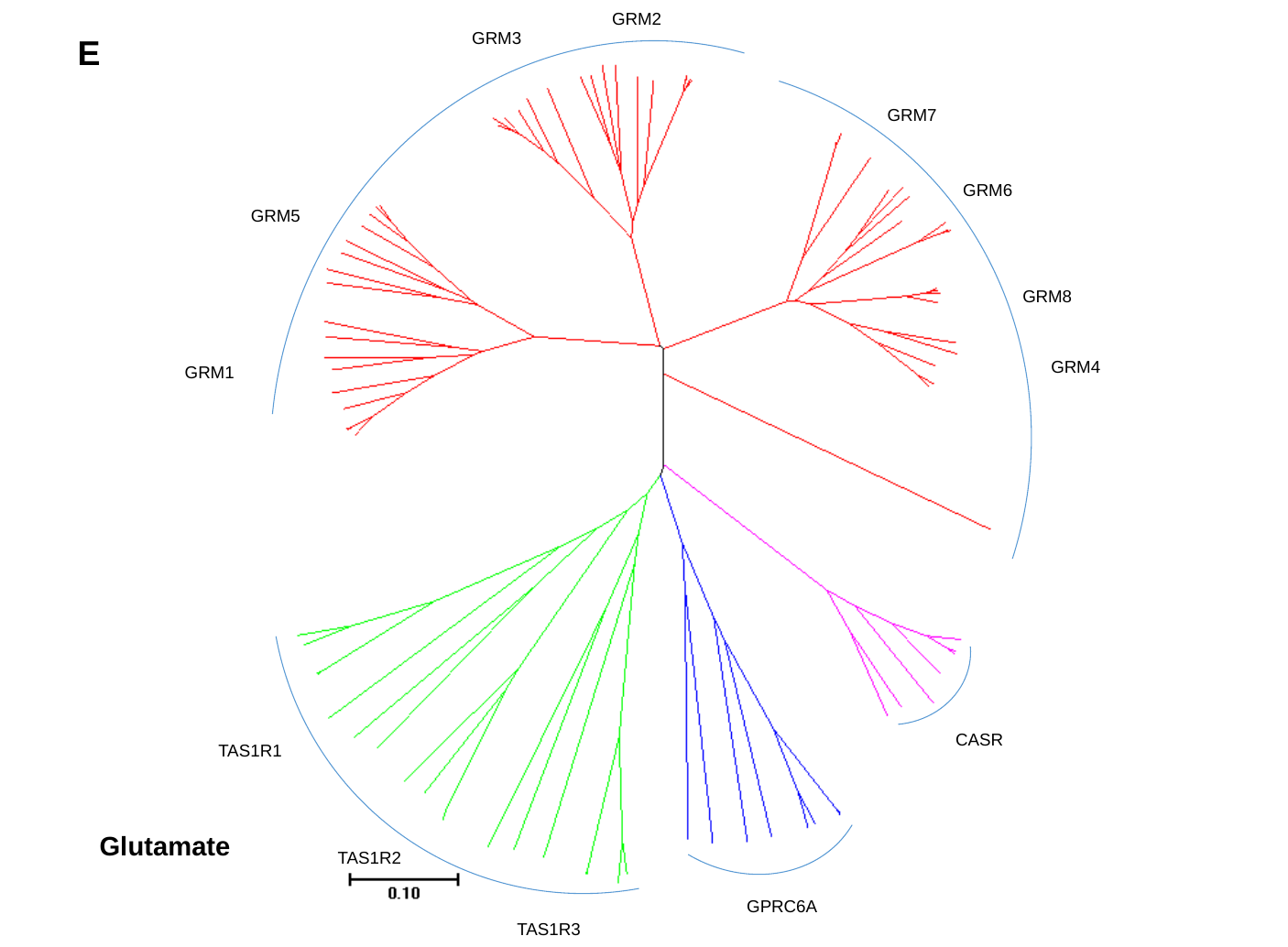

GRM2
GRM3
E
GRM7
GRM6
GRM5
GRM8
GRM4
GRM1
CASR
TAS1R1
Glutamate
TAS1R2
GPRC6A
TAS1R3

## Slide 6
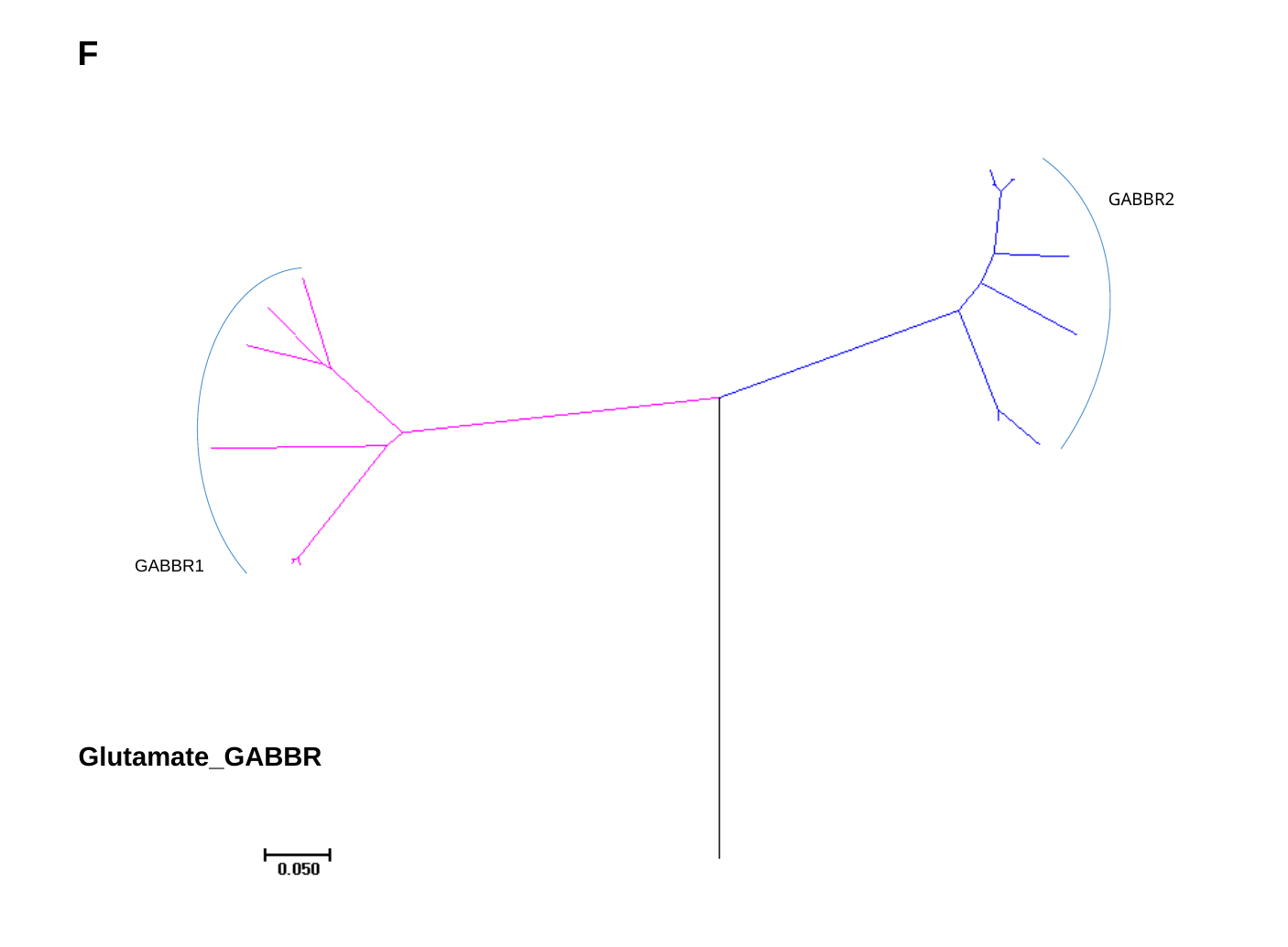

F
GABBR2
GABBR1
Glutamate_GABBR

## Slide 7
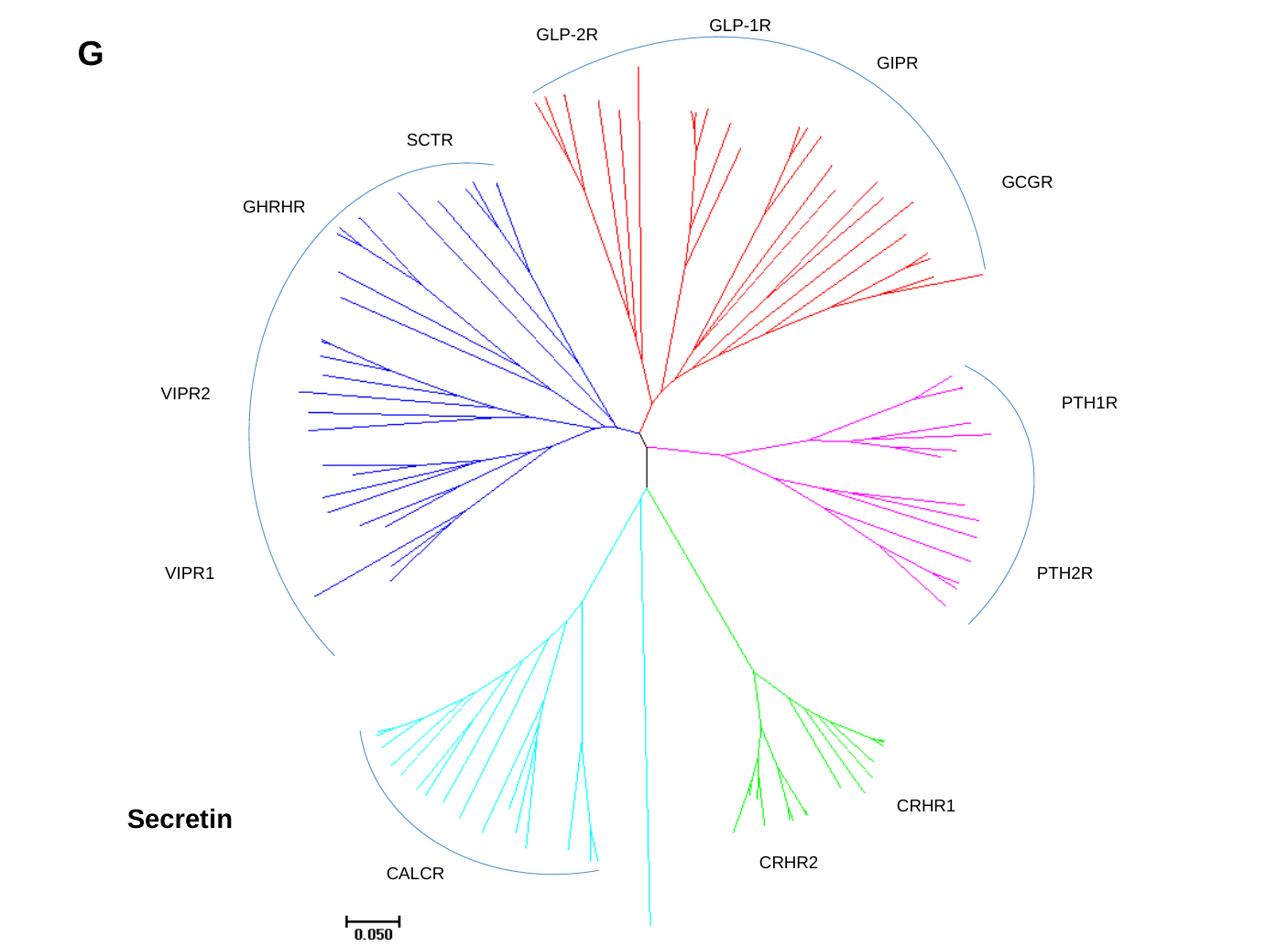

GLP-1R
GLP-2R
G
GIPR
SCTR
GCGR
GHRHR
VIPR2
PTH1R
VIPR1
PTH2R
CRHR1
Secretin
CRHR2
CALCR
