# Supplementary material for: Paralog-divergent Features May Help Reduce Off-target Effects of Drugs: Hints from Glucagon Subfamily Analysis
Source: Genomics Proteomics Bioinformatics. 2017 Jun 20;15(4):246–54. doi: 10.1016/j.gpb.2017.03.004 (PMC5582795; doi:10.1016/j.gpb.2017.03.004)
Supplement: Supplementary Table S1 — Type-I and type-II functionally-divergent residues in the binding sites of ligand and agonists to GLP-1R [file mmc4.docx]

**Table S1 Type-I and type-II functionally-divergent residues in the binding sites of ligand and agonists to GLP-1R**

| **Receptor residue** | **Location in receptor** | **Functional divergence** | **Ligand residue with direct interaction** |
| --- | --- | --- | --- |
| D198 | TM2 |  | H7 |
| K202 | TM2 |  | E9 |
| D293 | EC2 | Type II | H7, T11, F12, D15 |
| E294 | EC2 | Type I | T11 |
| G295 | EC2 |  | H7 |
| W297 | EC2 |  | F12 |
| N300 | EC2 |  | Y19 |

*Note*: TM, transmembrane domain; EC, extracellular domain.
