# Supplementary material for: Paralog-divergent Features May Help Reduce Off-target Effects of Drugs: Hints from Glucagon Subfamily Analysis
Source: Genomics Proteomics Bioinformatics. 2017 Jun 20;15(4):246–54. doi: 10.1016/j.gpb.2017.03.004 (PMC5582795; doi:10.1016/j.gpb.2017.03.004)
Supplement: Supplementary Table S2 — Variable residues in the binding sites of antagonist antibodies to GCGR [file mmc5.docx]

**Table S2 Variable residues in the binding sites of antagonist antibodies to GCGR**

| **Antagonist antibody** | **Alignment position** | **AA sequence** | | **AA conservation** |
| --- | --- | --- | --- | --- |
|  |  | **GCGR** | **GLP-1R** |  |
| mAb7 | 46 | 33FFFFFFKM | 36VVVVVVV | Variable sequence |
|  | 48 | 35KKKKKSSR | 38KKKKKKK | Variable sequence |
|  | 49 | 36WWWWWWWW | 39WWWWWWW | Conserved sequence |
|  | 50 | 37KKKKKKKM | 40RRRRRRK | Variable sequence |
|  | 52 | 39YYYYYYYY | 42YYYYYYY | Conserved sequence |
|  | 53 | 40GGGSSSTR | 43RRRRRRQ | Variable sequence |
|  | 54 | 41DDDDDEMD | 44RRRRRRD | Variable sequence |
|  | 88 | 72PPPPPLLL | 76EEPPSPL | Variable sequence |
|  | 90 | 74NNNNNNNN | 78GGGGGGG | Conserved property |
|  | 91 | 75TTTTTTTT | 79SSSSSST | Conserved property |
|  | 92 | 76TTTTTTTT | 80FFFFFFY | Variable sequence |
|  | 98 | 82PPPPPPPP | 86PPPPPPP | Conserved sequence |
|  | 99 | 83WWWWWWWW | 87WWWWWWW | Conserved sequence |
|  | 100 | 84YYYYYYYY | 88YYYYYYY | Conserved sequence |
|  | 101 | 85LLLLLLLL | 89LLLLLLL | Conserved sequence |
|  | 102 | 86PPPPPPPP | 90PPPPPPP | Conserved sequence |
|  | 103 | 87WWWWWWWW | 91WWWWWWW | Conserved sequence |
| mAb1 | 52 | 39YYYYYYYY | 42YYYYYYY | Conserved sequence |
|  | 81 | 65YYYYYFYY | 69YYYYYYY | Conserved property |
|  | 91 | 75TTTTTTTT | 79SSSSSST | Conserved property |
|  | 99 | 83WWWWWWWW | 87WWWWWWW | Conserved sequence |
|  | 100 | 84YYYYYFYY | 88YYYYYYY | Conserved property |
|  | 101 | 85LLLLLLLL | 89LLLLLLL | Conserved sequence |
|  | 103 | 87WWWWWWWW | 91WWWWWWW | Conserved sequence |
|  | 106 | 90KKKKKKQE | 94SSSSSST | Variable sequence |
|  | 110 | 94RRRRRRGG | 98GGGGGGG | Variable sequence |
|  | 114 | 98KKKKKKQV | 102RRRRRRR | Variable sequence |
|  | 122 | 106WWWWWWWY | 110WWWWWWW | Conserved property |
| mAb23 | 64 | 50LLLLLMQE | 54DDAAAAA | Variable sequence |
|  | 78 | 62FFFFFFFF | 66FFFFFFF | Conserved sequence |
|  | 81 | 65YYYYYFYY | 69YYYYYYY | Conserved property |
|  | 101 | 85LLLLLLLL | 89LLLLLLL | Conserved sequence |
|  | 103 | 87WWWWWWWW | 91WWWWWWW | Conserved sequence |
|  | 106 | 90KKKKKKQE | 94SSSSSST | Variable sequence |
|  | 110 | 94RRRRRRGG | 98GGGGGGG | Variable sequence |

*Note*: The multiple alignment is conducted using sequences of paralog GCGR and GLP-1R from human, chimpanzee, cow, mouse, rat, chicken, pig, fugu, and zebrafish. AA, amino acid.
